# An Alliance of Carbapenem-Resistant *Klebsiella pneumoniae* with Precise Capsular Serotypes and Clinical Determinants: A Disquietude in Hospital Setting

**DOI:** 10.1155/2022/6086979

**Published:** 2022-11-21

**Authors:** Elghar Soltani, Alka Hasani, Mohammad Ahangarzadeh Rezaee, Maryam Zaare Nahandi, Akbar Hasani, Pourya Gholizadeh

**Affiliations:** ^1^Immunology Research Center, Tabriz University of Medical Sciences, Tabriz, Iran; ^2^Clinical Research Development Unit, Sina Educational, Research and Treatment Center, Tabriz University of Medical Sciences, Tabriz, Iran; ^3^Department of Bacteriology and Virology, Faculty of Medicine, Tabriz University of Medical Sciences, Tabriz, Iran; ^4^Student Research Committee, Tabriz University of Medical Sciences, Tabriz, Iran; ^5^Department of Clinical Biochemistry and Laboratory Sciences, Faculty of Medicine, Tabriz University of Medical Sciences, Tabriz, Iran

## Abstract

Carbapenemase-resistant *Klebsiella pneumoniae* (CRKP) is a genuine burden for physicians and researchers. We aimed at carbapenemase resistance and its relation with capsular serotyping in *K*. *pneumoniae* and studied some clinical determinants, which may influence the clinical infections. Initially, 61 *K*. *pneumoniae* isolates obtained from various clinical specimens were confirmed at the molecular level and then antimicrobial susceptibility test was performed followed by capsular serotyping performed by multiplex PCR. All isolates were subjected to the detection of carbapenemase genes including *bla*_KPC_, *bla*_NDM-1_, *bla*_OXA-48_, *bla*_VIM_, and *bla*_IMP_. Clinical and demographic data of all patients were reviewed including age, gender, underlying diseases, and the treatment obtained. Multidrug-resistance was a predominant feature in 77% *K*. *pneumoniae* strains. Presence of extended-spectrum beta-lactamase was detected phenotypically in 59% *K*. *pneumoniae* strains. Carbapenem resistance was noticed phenotypically in 24.6% isolates. *bla*_OXA-48_ and *bla*_NDM-1_ were the most frequent carbapenemase genes. *bla*_NDM-1_ positive isolates correlated with gentamicin, amikacin, imipenem, and meropenem resistance (*p* < 0.05). The nosocomial isolates mostly harbored *bla*_OXA-48_ gene (*p* < 0.02). Amongst all the *K*. *pneumoniae* isolates, 59% isolates could be typed and serotype K54 had the highest prevalence followed by K20 and K5. Correlation between the carbapenemase genes, serotype and type of infection showed that *bla*_OXA-48_ positive strains had a significant association with K20 serotype and urinary tract infections (*p*=0.2) while, K20 serotype and *bla*_KPC_ positive strains were significantly associated with wound infections (K20, *p*=0.3 and *bla*_KPC_, and *p*=0.4). Mucoid phenotype was not found related to presence of specific carbapenemase genes or serotypes except serotype K20 (*p* < 0.001). Patients with monotherapy had treatment failure in comparison to the combination therapy for *bla*_KPC_-associated infections. In conclusion, the present investigation exhibited the significant association between K20 serotype with *bla*_OXA-48_. The predominance of K54 reveals the possibility of endemicity in our hospital setting. *K*. *pneumoniae* isolated from wound specimens significantly harbors K20 serotype and *bla*_KPC_ gene. Comprehensive clinical information and the distribution of antibiotic resistance genes, and serotypes may play important roles in the treatment process.

## 1. Introduction

*Klebsiella pneumoniae* has become more affluent in antibiotic resistance mechanisms and virulence features such that no one ever thought would come on too strong turning opportunistic bacteria into a potent pathogen. Clinical infections caused by this organism have become resistant to the treatment and increasingly life-threatening [1]. Acquiring the antibiotic resistance mechanisms, specifically, extended-spectrum *β*-lactamases (ESBLs) have rendered the bacteria resistant to cephalosporins and monobactams. Eventually, the emergence of carbapenemase [2] curbed the usage of imipenem and meropenem, thereby putting profound constraints on the therapeutic strategies [1]. In fact, the first case of *K*. *pneumoniae* expressing a carbapenemase was identified in 1996 and was named as KPC (*Klebsiella pneumoniae* carbapenemase) [3]. Later years witnessed an unprecedented increase in some additional enzymes inhibiting carbapenems, such as VIM (Verona integron-encoded Metallo-*β*-lactamase), IMP (Imipenemase), NDM (New Delhi Metallo-beta-lactamase) [4]. Enzymes IMP and VIM hydrolyze cephalosporins, penicillins, monobactam, and carbapenem except aztreonam [5], while NDM is a plasmid-borne [6] and OXA-48, the class *D β*-lactamase contains the carbapenemase activity which hydrolyzes imipenem and penicillin [7].

*K*. *pneumoniae* possesses several virulence treasures including a capsule, described as K types, which gives mucoid phenotype to the organism [8]. Though the organism has been discriminated into 79 capsular serotypes [9], nevertheless, the distribution of these types varies geographically and the type of infections.

To date, studies showing the correlation between antimicrobial resistance and serotypes of *K*. *pneumoniae* are limited worldwide, especially serotyping of carbapenem-resistant *K*. *pneumoniae*. In the past few years, emerging carbapenem-resistant hypervirulent *K*. *pneumoniae* (CR-hvKp) has become a serious threat for the treatment [10–12]. Moreover, the high potential dissemination of carbapenemase genes through serotype K1 have been reported in research studies [13, 14]. In this investigation, for the first time the dissemination of carbapenemase genes encoding the OXA-48, KPC, NDM-1, VIM, and IMP types were determined among six different capsular serotypes of *K*. *pneumoniae* strains. This prospective investigation covered various clinical infections, serotypes involved, and the presence of carbapenem-resistant genes to understand any relation amongst them in the context of *K*. *pneumoniae* infections. Host factors were given the insight to perceive the influence of them on the rise of antibiotic resistance.

## 2. Materials and Methods

### 2.1. Bacterial Isolates

The study was conducted on 61 *K*. *pneumoniae* clinical isolates obtained as a routine process in the Division of Microbiology, Sina Educational, Research and Treatment Center, Tabriz, Iran. Duplicate isolates from the same patient were not enrolled. In general, the inclusion criteria comprised of those *K*. *pneumoniae* isolates which were obtained as a pure isolate, the clinical manifestations of the patients matched with infectious conditions, and the infectious specialist suspected an infection. All clinical isolates were initially identified by conventional biochemical tests as described previously [15] and were confirmed by *K*. *pneumoniae* 16S–23S ITS (internal transcribed spacer) gene at the molecular level [16]. The isolates were defined phenotypically as mucoid when colonies were touched with a loop and a string-like growth was observed which adhered to the loop as it was lifted from the agar plate [17]. The pertinent information on any underlying disease, other demographic data, and the treatment regimens were collected from records of each patient. Response to the treatment of infection was assessed by the infectious disease specialist using clinical, biochemical, and microbiological parameters [18]. The identified strains were stored in tryptic soy broth containing 20% glycerol at −70°C for further experiments.

### 2.2. Antimicrobial Susceptibility Pattern

Antimicrobial susceptibility testing was performed using the Kirby–Bauer method in accordance with the Clinical and Laboratory Standard Institute (CLSI) guidelines [19]. The antibiotic disks including ciprofloxacin (5 *μ*g), amikacin (30 *μ*g), gentamicin (10 *μ*g), ceftazidime (30 *μ*g), cefotaxime (30 *μ*g), piperacillin-tazobactam (100/10 *μ*g), nitrofurantoin (300 *μ*g) (used only for urinary isolates), imipenem (5 *μ*g), meropenem (5 *μ*g), cotrimoxazole (1.25/23.75 *μ*g), and levofloxacin (5 *μ*g) were purchased from MAST-UK. *Escherichia coli* ATCC 25922 was used as quality control for antibiotic susceptibility testing. The combination disk diffusion test (CDDT) utilizing cefotaxime and ceftazidime with and without clavulanic acid was performed for the detection of ESBL production in *K*. *pneumoniae* isolates as per CLSI guidelines [20]. Resistance towards antibiotics belonging to at least three different antimicrobial classes was defined as multidrug resistance (MDR) [21].

### 2.3. DNA Isolation

The commercial DNA extraction kit (Stratec Biomedical systems, Birkenfeld, Germany) was used for the extraction of DNA from *K*. *pneumoniae* isolates. In brief, 1 mL of bacterial suspension matched equivalent to 0.5 McFarland was prepared from an overnight culture and then centrifuged. DNA was extracted as per the instructions provided in the kit from the pellet and finally resolved in 100 *µ*L TE buffer.

### 2.4. Analysis of the Carbapenemase Gene Regions

For PCR amplification of the carbapenemase genes, multiplex PCR was performed. The PCR reaction for *bla*_*KPC*_, *bla*_*NDM-1*_, *bla*_*OXA-48*_, *bla*_*IMP*_, and *bla*_*VIM*_ genes (total volume of 20 *μ*L) consisted 1× PCR buffer (20 mM Tris- HCl, 10 mM (NH_4_)2SO_4_, 1 mM KCl, mM MgSO_4_, and 0.1% Triton X-100), 2 Taq polymerase, 0.05 mM dNTP , and 50 *μ*mol/L primers for five targets (Yekta Tajhiz Azma®, Iran), as depicted in Table 1. PCR conditions for all five carbapenemase genes comprised of 35 cycles at 94°C for 5 min, 57°C for 40 sec, 72°C for 1 min, and final extension at 72°C for 7 min. Aliquots of the reaction mixtures were electrophoresed in 1.5% agarose gel (Yekta Tajhiz Azma, Iran) and stained with SYBR™ Safe DNA Gel Stain (Invitrogen).

### 2.5. Analysis of the Capsular Serotype Gene Regions

Capsular serotyping was performed using primers for the identification of K1, K2, K5, K20, K54, and K57 serotypes and PCR conditions described by Turton et al. [23] The amplified products were finally analyzed by electrophoresis in a 1% agarose gel (Yekta Tajhiz Azma®, Iran) run at 80 V for 1 h in 1×TBE buffer.

### 2.6. Statistical Methods

Statistical analysis was performed using descriptive statistics done by the *x*^2^ test and Fisher's exact test (if needed) to find the relationship between carbapenemase genes and other variables. Spearman's rank correlation was tested between carbapenemase genes and antibiotic resistance, mucoid phenotype, and hospital-acquired infection that were found to be statistically significantly correlation between values. Variables were analyzed using the SPSS statistics (version 20) program (IBM Corporation). All the tests were performed two sided and a *p* value ≤0.05 were considered statistically significant.

## 3. Results

### 3.1. Patient Information

Of 468 bacterial isolates isolated during four months, 61 (13.03%) were identified phenotypically as *K*. *pneumoniae*. Finally, these isolates were confirmed as *K*. *pneumoniae* at genetic level using the internal transcribed spacer region (*K*. *pneumoniae* 16S–23S) by polymerase chain reaction (PCR). These isolates were recovered from 33 (54.1%) females and 28 (45.9%) males. Age of the patients ranged from 3 to 89 years, with mean 56.7 ± 23.42 years, however, more than half clinical infections were seen in the elderly patients (>60 years). Thirty-four *K*. *pneumoniae* isolates (55.6%) were identified as nosocomial and 27 (44.4%) community-acquired pathogens. Forty-three (70.5%) isolates were obtained from in-patients and 11 (18%) from outpatients. Forty-three (70.5%) isolates were associated with patients who live in the city, whilst 18 (29.5%) encompassed rural areas.

### 3.2. Clinical Source

Clinical source of these 61 isolates comprised of urine (*n* = 31; 50.8%), wound (*n* = 15; 24.6%), blood (*n* = 8; 13.1%), endotracheal aspirates (*n* = 4; 6.6%), and other body fluids (*n* = 3; 4.9%). These isolates were collected from the patients admitted to the intensive care unit (ICU) (36.2%) followed by internal (13.1%), burn (9.8%), urology (9.8%), infectious (6.6%), and emergency (3.3%) wards.

### 3.3. Antibiotic Resistance

In this study, prevalence of antimicrobial resistance markers was relatively high for drugs used as traditional therapy in the treatment of UTIs, such as nitrofurantoin (68.9%), ciprofloxacin (68.9%), and cotrimoxazole (67.2%). Out of the total 61 analyzed isolates, 78.8% and 75.4% isolates were resistant to cefotaxime and ceftazidime, respectively, and of these, 36 (59%) were positive for extended-spectrum beta-lactamase (ESBL) production by the double-disk synergy test. Resistance towards other antibiotics was as follows: piperacillin-tazobactam (57.4%), gentamicin (45.9%), amikacin (39.3%), levofloxacin (24.6%), imipenem (24.6%), and meropenem (24.6%). Forty-seven (77%) *K*. *pneumoniae* isolates were found as MDR, while 6 (9.8%) retained their susceptibility to all classes of antibiotics tested.

### 3.4. Capsular Serotyping

Capsular typing performed at a molecular level could type 36 (59%) *K*. *pneumoniae* isolates. Serotype K54 had the highest prevalence (*n* = 18; (29.5%)) followed by K20 (*n* = 13; (21.3%)) and K5 (*n* = 5; (8.1%)). We did not observe K1, K2, and K57 capsular serotypes in the present investigation.

### 3.5. Prevalence of Carbapenemase Genes

Prevalence of *bla*_OXA-48_, *bla*_KPC_, and *bla*_NDM-1_ genes among 61 *K*. *pneumoniae* isolates was 48 (78.7%), 9 (14.7%), and 12 (19.6%), respectively. The *bla*_VIM_ and *bla*_IMP_ genes had lower prevalence (7 (11.4%) and 3 (4.9%), respectively). Seven (11.4%) *K*. *pneumoniae* did not furnish any carbapenemase gene.  Table 2 provides the distribution of K-serotypes, carbapenemase genes in 61 *K*. *pneumoniae* isolates. The presence of *bla*_OXA-48_ was highly associated with K20 positive isolates (*p*=0.2). In contrast, other carbapenemase genes could not be related to any of the capsular serotypes.  Table 3 shows when hospital-acquired *K*. *pneumoniae* isolates were compared for carbapenemase genes, 31 of the 48 *bla*_OXA-48_ positive isolates had a significant (*p* < 0.002) relation with hospital-acquired isolates. On the contrary, *bla*_NDM-1_, *bla*_KPC_, *bla*_VIM_, and *bla*_IMP_ positive isolates were not related to hospital-acquired isolates ((*p*=0.500), (*p*=0.607), (*p*=0.406), and (*p*=0.438), respectively).

### 3.6. Mucoid Phenotype

Sixteen (26.22%) *K*. *pneumoniae* isolates appeared as mucoid phenotype in the following clinical specimens: body fluid (*n* = 2; 12.5%), blood (*n* = 2; 12.5%), wound (*n* = 5; 31.2%), and urine (*n* = 7; 43.7%). Among 16 mucoid phenotype isolates, 14 were found as typeable (87.5%; *p*=0.01). Among capsular serotypes, in correlation with mucoid phenotype, 10 of 13 K20 positive isolates have mucoid phenotype (*p* < 0.001), 6 of 18 K54 positive isolates (*p*=0.304), and 1 of 5 K5 positive isolates (*p*=0.606) showed mucoid phenotype, while none of the carbapenemase genes had a significant relationship with mucoid phenotype (Table 3).

### 3.7. Correlation between Carbapenemase Genes and Antibiotic Resistance

**Figure 1:**
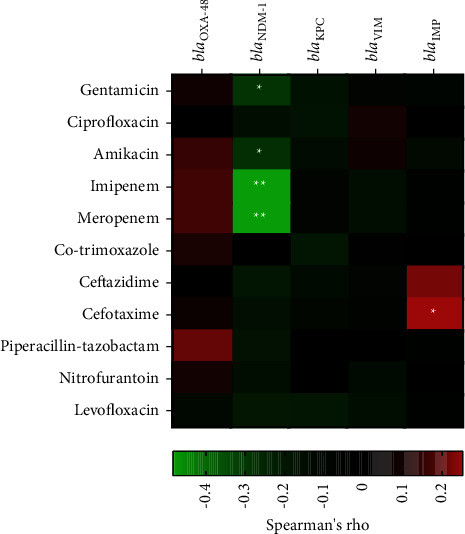
Correlation between carbapenemase genes and antibiotic resistance. Correlation was tested using Spearman's rank test. Spearman's rho values are shown the color of each correlation test corresponding as heatmaps. ^*∗*^*p* value <0.05, ^*∗∗*^*p* value <0.01.

Figure 1 displays the comprehensive correlation between carbapenemase genes and antibiotic resistance. Among carbapenemase genes, significant correlations were found between *bla*_NDM-1_ and gentamicin (*r* = -0.289, *p* value <0.05), amikacin (*r* = -0.277, *p* value <0.01), and imipenem and meropenem (*r* = -0.484, *p* value <0.01). Another significant correlation was found between *bla*_IMP_ and cefotaxime resistance (*r* = 0.252,*p* value <0.05).

### 3.8. Prevalence of Carbapenemase Genes in Predominant Serotypes

**Figure 2:**
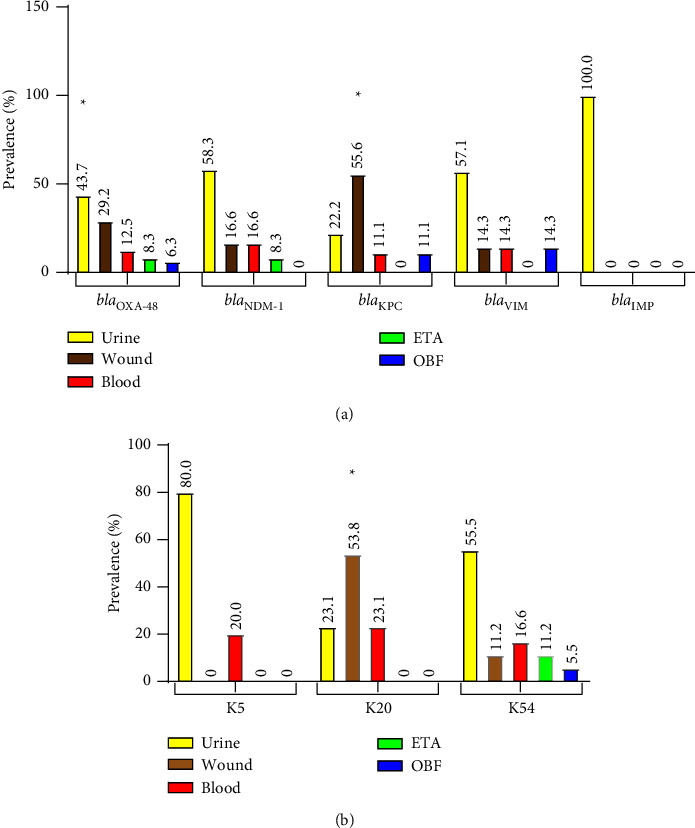
Prevalence of capsular serotypes and carbapenemase genes among various clinical specimens. (a) Prevalence of carbapenemase genes. (b) Prevalence of capsular serotypes ETA: endotracheal aspirate; OBF: other body fluid; (K) capsular polysaccharide (K antigen). ^*∗*^*p* value <0.05.

Figure 2 depicts the information on the distribution of carbapenemase genes and capsular serotypes among the various clinical specimens. Most of the isolates obtained from the wound specimens belonged to K20 serotype (*p*=0.03) and harbored *bla*_KPC_ gene (*p*=0.04) in their genome. On the other hand, *bla*_OXA-48_ positive *K*. *pneumoniae* strains were mostly isolated from urine specimens (*p*=0.02). In the present investigation, carbapenemase gene *bla*_OXA-48_ was observed in *K*. *pneumoniae* isolates obtained from patients admitted to internal and infectious wards; however, this gene was strongly associated with K20 serotype in *K*. *pneumoniae*-infected patients admitted to burn wards including burn ICU, especially in-patients who developed *K*. *pneumoniae* infections after the grafting procedure (Table 4).

### 3.9. Clinical Data

The clinical manifestations of the 61 patients with *K*. *pneumoniae* infections comprised of renal diseases (*n* = 20; 32.7%), pulmonary diseases (*n* = 11; 18%), 7 (11.4%) had infectious diseases, 6 (9.7%) suffered from ulcers and abscess, 10 (16.4%) were burn patients, 5 (8.2%) patients were diabetic, and 2 (3.3%) had hyperplasia of prostate. Nine (14.8%) patients underwent mechanical ventilation. The patient's record yielded the information pertinent to the treatment strategies. Among 50 in-patients, 41 cases were prescribed cephalosporins, 27 cases received a fluoroquinolone, 11 cases were treated with a carbapenem, 10 cases received an aminoglycoside, and 3 cases received tetracycline. Ciprofloxacin (*n* = 27; (54%)) was the most commonly used antibiotic against *K*. *pneumoniae* infections in this study and cefixime was only used for the treatment of one (2%) patient. Overall, 20 (40%) cases received treatment as monotherapy, while 30 (60%) patients received combination therapy.

### 3.10. Treatment Failure

The overall rate of treatment failure in the present study was 28%. Wound infections were associated with the highest rate of treatment failure (*p*=0.03). Almost 55% of KPC-associated *K*. *pneumoniae* infections failed to respond to conventional therapeutic regimens. Also, 44.4% of K54 serotype-associated infections had treatment failure with ciprofloxacin (*p*=0.02). When mortality was analyzed in relation to the infections caused by *K*. *pneumoniae* infections, overall mortality was 11 (18%) among patients in our hospital. In total, 55.5% of KPC positive isolates were involved in the mortality rate (*p*=0.007).

## 4. Discussion

*K*. *pneumoniae* has become notorious for causing nosocomial and difficult to treat clinical infections. In this study, prevalence of hospital-acquired *K*. *pneumoniae* infections was 55.6%. Our record is higher than other nosocomial infections reported earlier from Iran, Turkey, and Southern Europe (23.5%) [24], which may be due to diverse infections analyzed in medical practices at different countries. Similar to other research studies [25, 26], *bla*_OXA-48_ was the most prevalent (78.8%) carbapenemase factor in the present research followed by *bla*_NDM-1_. Seven (11.47%) *K*. *pneumoniae* isolates produced both *bla*_OXA-48_ and *bla*_NDM-1_. Coproduction of *bla*_*OXA-48*_ and *bla*_*NDM-1*_ carbapenemase genes have been reported in the investigations conducted in Turkey [22] and the United States [27]. The coexistence of these two carbapenemase genes in a pathogen constrains the treatment options for the clinicians and potential for global dissemination by means of cross-border transfer [28]. It is apparent that carbapenem resistance is on move and has increased comparatively since last five years. The current study was in concordance with other investigations conducted on imipenem resistance in Brazil and New York [29, 30]. There exist a variation in prevalence of imipenem resistance geographically as well as the usage of antibiotics. Investigations carried out recently in Iran and earlier in New York [5, 29], respectively, reported higher resistance to imipenem while Indian research found much lower resistance [31].

The present investigation found high prevalence of carbapenemase genes which is compatible with another US study where one-third of gross *K*. *pneumoniae* isolates carried the carbapenemase enzymes [29]. Prevalence of the *bla*_VIM_ genes in our study was 11.4% which is though lower than the two studies conducted in Canada and the US [32, 33] earlier. These differences may be due to the plasmid-born *bla*_VIM_ gene and the genetic diversity among strains. IMP-type enzymes are one of the major groups of MBLs [34], encoded on plasmid and integrons and thus, spread easily [35] but they have low prevalence comparatively [22, 33]. The current study observed *bla*_IMP_ gene merely in 4.91% isolates.

*K*. *pneumoniae* carbapenemase (KPC) associated with *K*. *pneumoniae* infections are predominantly nosocomial and systemic. These type of infections are frequently encountered in patients possessing multiple risk factors [36]. Similar to a study conducted on the bacteremic patients for the outcome of the treatment [37], we observed that 55.5% of *bla*_KPC_ positive isolates were associated with mortality. Both monotherapy and combination therapy regimens are used for the treatment of KPC infections in our hospital. The present study observed that 60% of expired patients were infected with KPC-producing isolates and had received monotherapy. Lee and Burgess [18] concluded in their study that combination therapy should be considered for the treatment of KPC infections as monotherapy lead to higher rates of treatment failure.

Some capsular serotypes including K1, K2, K5, K16, K20, K54, K57, and KN1 are known as hyper virulent variants of *K*. *pneumoniae* [9]. The results of our study did not find much variability in the presence of serotypes. In the current study, K54 was the most frequent (29.5%) serotype followed by K20 (21.3%) and K5 (8.2%). In total, 36 (59%) isolates were typeable. Differences are observed when seroepidemiology is compared geographically thus, knowledge of the existing serotypes is mandatory. A study conducted on the frequencies of capsular serotypes among 703 *Klebsiella* isolated from the blood of hospitalized patients found more than 90% of the isolates typeable [38], while another research from Taiwan, despite the inclusion of the high number of isolates, could not type the isolates enormously [39]. A recent study reported from Iran found K54 as the most frequent (68%) capsular serotype while K1 (8%) had the lowest frequency [40]. Prevalence of K5, K20, and K54 serotypes is significantly lower in Europe and Taiwan in comparison to our study [38, 41]. High prevalence of serotypes K54 and K20 in our *K*. *pneumoniae* isolates requires medical attention.

About 26.22% of *K*. *pneumoniae* isolates in this study showed a mucoid phenotype that was associated with capsular serotypes other than K1 and K2. In a study conducted by Victor et al., similar to our work, about 24% of isolates with serotypes other than K1 and K2 had a mucoid phenotype, while a higher percentage of mucoid phenotype was associated to serotypes K1 and K2 [17].

A study in Uganda accomplished by Ssekatawa et al., found that serotypes K1, K2, K5, and K20 were identified among *K*. *pneumoniae* isolates and were not belonged to serotypes K54 and K57. In addition, they reported which carbapenemase resistance genes were identified among 16/42 serotype K5 and in 11/35 serotype K20 [42]. Nevertheless, comparison of carbapenem resistance among the K-serotypes showed chi square *p* values >0.05 indicating insignificant correlation between them. Similarly, in this survey, 4 of 5 serotype K5, all serotype K20, and 15 of 18 serotype K54 harboring carbapenemase genes. We assessed the relationship between capsular serotypes and the presence of carbapenemase genes. Ours is the first of its kind research study which showed serotype K20 to be associated with the presence of *bla*_OXA-48_-mediated carbapenem resistance (*p* < 0.05). It is noteworthy that five *K*. *pneumoniae* isolates with the same antibiotic resistance profile (GM, CIP, AN, SXT, CAZ, CTX, PTZ, and NI) belonged to K20 serotype and all had acquired plasmid containing *bla*_OXA-48_ gene. According to our findings, serotype K20 was associated with amikacin and gentamicin resistance while ciprofloxacin-resistant isolates belonged to serotype K54. In addition, NDM-1 had a high prevalence in MDR isolates with resistance against aminoglycosides and carbapenems (*p* < 0.05). Our study is similar to Flores et al. research conducted in the year 2020. They characterized NDM-producing *K*. *pneumoniae* isolates and found that this gene was mostly detected in MDR, PDR, and XDR isolates and also described the coexistence of NDM-producing *K*. *pneumoniae* with other carbapenemase genes such as *bla*_OXA-48_, *bla*_VIM_, and *bla*_KPC_ [43]. Ciprofloxacin, which is the most common antibiotic prescribed for the treatment of *K*. *pneumoniae* infections in our hospitals, should also be given a second thought as the most frequent serotype K54 isolates were characterized belonging to ciprofloxacin-resistant.

Acquisition of *bla*_OXA-48_-mediated carbapenem resistance with intimacy with serotype K20 was a general feature in *K*. *pneumoniae*-infected patients admitted to burn wards including burn ICU, especially in-patients who developed *K*. *pneumoniae* infections after the grafting procedure. However, *bla*_OXA-48_ positive *K*. *pneumoniae* were also isolated from in-patients admitted to the internal ward. Extension of *bla*_OXA-48_ gene coding epidemically significant carbapenemase among hospital pathogens is important for the regional and global epidemiology of antimicrobial resistance [44]. We also observed that urine and wound specimens had the largest number of bacteria carrying carbapenemase-encoding gene with *bla*_OXA-48_ gene associated with urinary tract infections while *bla*_KPC_ was mostly positive in wound infections. These findings are similar to those observed in African countries [45–47]. Earlier an association of KPC positivity and bacteremia has been witnessed with higher mortality [37].

In this investigation, a high percentage (19.6%) *K*. *pneumoniae* isolates carried *bla*_NDM-1_ gene. NDM-harboring Gram-negative strains are known as a serious public health concern [48]. The *bla*_NDM-1_ positive *K*. *pneumoniae* isolates with the multidrug-resistant feature could quickly disseminate all around the world and create an alarming risk situation. Moreover, a large 180-kb plasmid specialized for *K*. *pneumoniae* which possesses *bla*_NDM-1_ gene has high potential for transfer to susceptible *E. coli* J53 at a high frequency. Also, multiple resistance genes are located on this plasmid which create resistance against almost all antibiotics. As *bla*_NDM-1_ gene has potential to spread rapidly among clinically relevant bacteria, it may lead to a severe threat in therapeutics [48]. A previous report suggested a high attention to colonization pressure and the infection prevention control strategies for minimizing the rapid dissemination of *bla*_NDM-1_ harboring plasmids in specified geographical areas [49].

## 5. Conclusion

To the best of our knowledge, this is the first study performed on *K*. *pneumoniae* strains isolated from Northwest Iran, aiming to investigate the correlations among the capsular serotypes, carbapenem resistance, and the clinical determinants involved. This study highlighted a high prevalence of carbapenem-resistant genes in *K*. *pneumoniae* isolates. Of the five carbapenemase genes studied, the association of *bla*_OXA-48_ was observed in serotype K20 isolates. *bla*_OXA-48_ positivity was correlated with patients afflicted with urinary tract infections with the hospital as a source. *bla*_KPC_ positive strains and K20 serotype were significantly associated with wound infections. Appropriate information regarding the distribution of antibiotic resistance genes, serotypes, and other characteristic features in relation to the specific clinical specimens and medical wards could help physicians to choose the appropriate treatment.

## Figures and Tables

**Table 1:** Carbapenemase gene regions [22].

Genes	Primers	Sequences (5′-3′)	Product sizes (bp)
*bla*_OXA-48_	OXA-48-F	5′-GCGTGGTTAAGGATGAACAC-3′	438
	OXA-48-R	5′-CATCAAGTTCAACCCAACCG-3′	
*bla*_NDM-1_	NDM-1-F	5′-GGTTTGGCGATCTGGTTTTC-3′	621
	NDM-1-R	5′-CGGAATGGCTCATCACGATC-3′	
*bla*_KPC_	KPC-F	5′-CGTCTAGTTCTGCTGTCTTG-3′	798
	KPC-R	5′-CTTGTCATCCTTGTTAGGCG-3′	
*bla*_VIM_	VIM-F	5′-GATGGTGTTTGGTCGCATA-3′	390
	VIM-R	5′-CGAATGCGCAGCACCAG-3′	
*bla*_IMP_	IMP-F	5′-GGAATAGAGTGGCTTAAYTCTC-3′	232
	IMP-R	5′-GGTTTAAYAAAACAACCACC-3′	

**Table 2:** Distribution of carbapenemase genes in predominant capsular serotypes.

Capsular serotypes	Carbapenemase genes				
	*bla*_OXA-48_ (*n* = 48)	*bla*_*NDM-1*_ (*n* = 12)	*bla*_*KPC*_ (*n* = 9)	*bla*_*VIM*_ (*n* = 7)	*bla*_*IMP*_ (*n* = 3)
K5 (*n* = 5)	3	1	1	1	0
K20 (*n* = 13)	13^*∗*^	2	3	1	1
K54 (*n* = 18)	13	5	3	3	2

K: capsular polysaccharide (K antigen). ^*∗*^*p* value <0.05, calculated by chi-squared test or Fisher's exact test.

**Table 3:** Correlation of carbapenemase genes with mucoid phenotype and hospital-acquired infection among *K*. *pneumoniae* isolates.

Variables	Number. (Spearman's rho) of				
	Carbapenemase genes				
	*bla*_OXA-48_ (*n* = 48)	*bla*_NDM-1_ (*n* = 12)	*bla*_KPC_ (*n* = 9)	*bla*_VIM_ (*n* = 7)	*bla*_IMP_ (*n* = 3)
Mucoid phenotype (*n* = 16)	15 (*r* = 0.219)	2 (*r* = -0.108)	1 (*r* = -0.143)	2 (*r* = 0.019)	0 (*r* = -0.136)
Hospital-acquired infection (*n* = 34)	31 (*r* = 0.404)^*∗∗*^	7 (*r* = 0.042)	5 (*r* = 0.012)	3 (*r* = -0.081)	1 (*r* = -0.095)

Correlation was tested using Spearman's rank test. ^*∗∗*^*p* value <0.01.

**Table 4:** Distribution of carbapenemase genes between various clinical wards.

Wards	Number of					
	*K. pneumoniae*, *n* (%)	Carbapenemase genes				
		*bla*_OXA-48_ (*n* = 48)	*bla*_NDM-1_ (*n* = 12)	*bla*_KPC_ (*n* = 9)	*bla*_VIM_ (*n* = 7)	*bla*_IMP_ (*n* = 3)
Burn ICU	7 (11.5)	8^*∗*^	2	1	0	0
General ICU	6 (9.8)	4	1	1	0	0
Infectious ICU	4 (6.6)	3	0	2	1	0
Internal ICU	3 (4.9)	2	1	1	0	0
Surgery ICU	2 (3.3)	2	0	0	0	1
Internal	8 (13.1)	7^*∗*^	0	1	2	1
Burn	6 (9.8)	6^*∗*^	1	1	2	0
Urology	6 (9.8)	3	2	0	1	0
Surgery	2 (3.3)	2	0	1	0	0
Emergency	2 (3.3)	1	1	0	0	0
Infectious	4 (6.6)	4	2	0	0	0
Out- patients	11 (18)	6	2	1	1	1

ICU: intensive care unit ^*∗*^*p* value <0.05, calculated by chi-squared test or Fisher's exact test.

## Data Availability

The authors declare that the data used to support the findings of this study are available from the corresponding author upon request.
